# Polyethylene Nanoplastics Intensify Arsenic Toxicity in Lettuce by Altering Arsenic Accumulation and Stress Pathways

**DOI:** 10.3390/toxics14030266

**Published:** 2026-03-18

**Authors:** Mengyuan Wang, Weijie Qin, Yue Zhang, Weixin Fan, Li Mu, Junxing Li, Lihong Dai, Chunsheng Qiu

**Affiliations:** 1 Tianjin Key Laboratory of Agro-Environment and Safe-Product, Key Laboratory for Environmental Factors Control of Agro-Product Quality Safety (Ministry of Agriculture and Rural Affairs), Institute of Agro-Environmental Protection, Ministry of Agriculture and Rural Affairs, Tianjin 300191, China; tengweimeng@gmail.com (M.W.); qwj13722689216@163.com (W.Q.); zy121065@163.com (Y.Z.); ljx77-77@126.com (J.L.); dailihong81@126.com (L.D.); 2 Tianjin Key Laboratory of Aqueous Science and Technology, School of Environmental and Municipal Engineering, Tianjin Chengjian University, Tianjin 300384, China; qcs254@163.com; 3 Xinjiang Key Laboratory of Agricultural Green Materials and Soil Health, Institute of Western Agricultural of CAAS, Changji 831100, China

**Keywords:** arsenic accumulation, soil plant transfer, nanoplastic-enhanced arsenic toxicity, metabolomic profiling, agricultural soil

## Abstract

Nanoplastics (NPs) are increasingly detected in agricultural soils, yet their influence on arsenic (As) transfer and plant toxicity remains unclear. Lettuce (*Lactuca sativa* L.) was cultivated in farmland soil with a naturally high As background (98.8 mg·kg^−1^) to assess how polyethylene nanoplastics (PE NPs) affect rhizosphere conditions, As accumulation, and plant performance. PE NPs partially buffered soil acidification but reduced rhizosphere water content, while total soil As remained largely unchanged. Leaf As increased by 35–39%, with reduced biomass (up to 30%) and lower chlorophyll status (SPAD ~7% lower). Metabolomic analyses indicated dose-dependent alterations in central carbon metabolism and phenylalanine-related antioxidant metabolites, including suppressed tricarboxylic acid cycle intermediates at higher PE levels. Overall, PE NPs enhanced transfer of background As to edible leaves and intensified phytotoxicity, underscoring the need to consider nanoplastics in risk assessment of As-affected soils.

## 1. Introduction

Global plastic production remains massive due to the low cost and wide applicability of these materials. After disposal, plastic waste fragments and weathers, generating persistent microplastics and nanoplastics (NPs) in the environment [1,2]. In agricultural soils, primary sources of NPs include plastic mulching, wastewater irrigation, and the application of organic amendments [3,4]. Once incorporated into the soil, NPs modify key physicochemical properties including pH, soil aggregation, and water retention [4,5]. NPs also influence microbial communities and nutrient cycling, which subsequently affects plant water uptake and stress responses. Although NPs have been intensively studied in aquatic systems, their behaviour in agricultural soils remains unclear, particularly in the presence of other contaminants [6,7].

Arsenic (As) is a toxic metalloid that threatens crop production and food safety. In southern China, farmland soils frequently exhibit elevated As levels. This contamination results from natural geochemical enrichment and anthropogenic activities, such as mining and wastewater irrigation [1,8]. Upon uptake by roots, As accumulates in edible tissues. This accumulation subsequently induces oxidative stress, metabolic disruption, and growth inhibition [8]. Plant resistance depends on As speciation, soil properties and physiological traits, which collectively govern As uptake and detoxification efficiency [5]. Previous studies have shown that arsenic exposure disrupts central carbon and nitrogen metabolism, leading to coordinated shifts in energy production, redox balance and antioxidant defence. Such alterations occur at the pathway and network levels rather than being confined to individual physiological parameters [9]. Metabolomics has therefore been widely applied to characterize system-wide metabolic reprogramming under environmental stress because it enables detection of coordinated alterations across interconnected biochemical pathways [9]. However, whether nanoplastic exposure modifies arsenic-induced metabolic regulation and alters the balance between adaptive adjustment and metabolic impairment remains unclear.

Recent studies indicate that NPs act as active modulators of co-occurring contaminants rather than passive background pollutants. Nanoparticles (NPs) can adsorb arsenic at the soil–solution interface and act as co-transport carriers due to their high specific surface area and functionalizable surfaces. Additionally, they may indirectly regulate arsenic morphology, mobility, and plant toxicity by influencing microbial processes (e.g., mycorrhizal fungal activity) and plant physiological responses [10,11,12]. Such interfacial interactions can influence the partitioning of arsenic between solid and liquid phases. Within the rhizosphere, NPs further affect redox conditions and the distribution of water, thereby altering the microenvironment through which arsenic is transported towards roots [13]. Previous experiments demonstrate that microplastics and nanoplastics enhance As accumulation in plants and intensify oxidative stress responses [3]. Among these materials, polyethylene is a dominant component of agricultural mulching films. Extensive production has led to the ubiquitous presence of PE fragments in farmland soils [14]. Given that nanoplastics are commonly defined as plastic particles smaller than 1000 nm and that particles at the nanoscale exhibit size dependent surface reactivity and biological interactions, PE NPs with a nominal size distribution of 80 to 120 nm were selected as a controlled nanoscale model for mechanistic investigation [15,16]. Lettuce (*Lactuca sativa* L.) serves as an ideal model for assessing toxicity in soil due to its short growth cycle and high sensitivity to contaminants [17].

While previous studies suggest that plastics modify As mobility, the mechanisms driving As transfer to edible tissues under realistic soil conditions remain obscure. To address this knowledge gap, this study employed lettuce to investigate the impact of PE NPs on As behaviour in agricultural soil with naturally high background As levels. Specifically, the study aimed to quantify the effects of PE NPs on soil pH and water content by PE NPs, evaluate consequent changes in As uptake and biomass production, and elucidate dose-dependent metabolic shifts. By integrating soil physicochemical data with physiological and metabolomic profiles, this study clarifies the mechanisms by which PE NPs intensify As toxicity. These insights provide a mechanistic basis for assessing risks associated with plastic residues in contaminated agroecosystems.

## 2. Materials and Methods

### 2.1. Reagents and Materials

The experimental soil was collected from the surface layer (0–20 cm) of farmland in Hainan Province, with a total arsenic concentration of 98.756 mg·kg^−1^, which is 3.29 times higher than the national standard limit of 30 mg·kg^−1^, according to Soil Environmental Quality—Risk Control Standard for Soil Contamination of Agricultural Land (GB15618-2018) [18]. After collection, the soil was air-dried at room temperature, manually cleared of visible stones and plant roots, and then passed through a 2 mm mesh prior to use. PE NPs (Qingdao Abate Instrument Technology Co., Ltd., Qingdao, China) had a nominal particle size distribution of 80–120 nm according to the manufacturer’s specifications. Lettuce (*Lactuca sativa* L.) seeds (Zhonglv Agriculture Group Co., Ltd., Xiamen, China) were selected to ensure uniform size.

### 2.2. Experimental Design and Exposure Treatments

A pot experiment was conducted using soil with a naturally high arsenic background to assess the effects of PE NPs on lettuce. The background arsenic concentration of the soil was 98.8 mg kg^−1^. The collected soil was sieved through a 20 mesh screen, and 5 kg of soil was placed into each pot. PE NPs were thoroughly mixed with soil at the designated concentrations prior to seedling transplantation. The amended soils were pre incubated for 15 days to allow equilibration between nanoplastics and soil, with manual mixing every three days to ensure homogeneous distribution. Three treatments were established: (i) S group, soil without nanoplastics; (ii) SP1 group, soil amended with 10 mg kg^−1^ PE nanoplastics; and (iii) SP2 group, soil amended with 100 mg kg^−1^ PE nanoplastics. Lettuce seeds were sterilized with 75 percent ethanol for 3 min, rinsed three times with ultrapure water, soaked overnight, and germinated under greenhouse conditions maintained at 15 to 25 °C and 50 to 70 percent relative humidity. Uniform seedlings were transplanted into the prepared pots. Each pot contained one lettuce plant. Three independent pots were established for each treatment. Samples were collected at 15, 30 and 45 days after transplantation. At each sampling time, plants were carefully uprooted from the pots. Loosely attached soil was gently shaken off and collected as non-rhizosphere soil. The soil that remained tightly adhered to the root surface after gentle shaking was carefully brushed off and defined as rhizosphere soil. Both soil compartments were immediately stored for subsequent physicochemical analyses.

### 2.3. Soil Physicochemical Properties and Arsenic Analysis

Arsenic was quantified in both soil and plant samples. In this study, the total arsenic content in soil was analyzed in accordance with the provisions of the GB15618-2018. The total arsenic content in soil was determined following microwave digestion and atomic fluorescence spectrometry in accordance with HJ 680-2013 [19]. Approximately 0.10 g of air-dried soil was digested using hydrochloric acid and nitric acid under controlled microwave heating conditions. After digestion and cooling, the solution was diluted to a fixed volume with ultrapure water and analyzed using an atomic fluorescence spectrometer (SA 50 LC AFS, Jitian Instruments, Beijing, China). According to GB15618-2018, the total arsenic content in soil was analyzed in this study.

The arsenic content in plant was determined by ICP-MS following a validated extraction protocol widely used in plant arsenic analysis. Fresh leaf samples (0.5 g) were thoroughly washed with ultrapure water, frozen in liquid nitrogen and ground to a fine powder. The powder was extracted with 10 mL of a solution containing 2 mmol·L^−1^ ammonium phosphate dibasic and 0.2 mmol·L^−1^ disodium EDTA, shaken at 250 r·min^−1^ for 20 min and centrifuged at 10,000 r·min^−1^ for 5 min, following the method described by Dong et al. (2020) [6]. The supernatant was filtered through 0.45 μm membranes and analyzed for total arsenic by inductively coupled plasma mass spectrometry (ICP MS). Each batch of analyses included at least one reagent blank and two certified reference materials, GBW(E)100348 and GBW10049 (GSB 27), to ensure measurement accuracy and data reliability.

Soil physicochemical properties were analyzed for both rhizosphere and non-rhizosphere soils. Soil pH was measured at a soil-to-water ratio of 1:2.5 using a glass electrode pH metre [20]. This approach is widely applied in agricultural soil assessment to evaluate active acidity directly relevant to plant growth and rhizosphere processes [14]. Soil water content was determined gravimetrically by drying pre-weighed moist soil samples at 105 °C to constant weight. Moisture content (%) was calculated as

$$Moisture\;content\left(\%\right)=\frac{M_{0}-M_{1}}{M_{0}}*100\%$$

where *M*_0_ is the soil weight before drying and *M*_1_ is the soil weight after drying.

### 2.4. Physiological Indices and Metabolomic Analysis of Lettuce

To evaluate the overall effects of different treatments on lettuce, growth performance, antioxidant status, nutritional indices and leaf metabolomic profiles were measured. Growth parameters, including stem length, root length, leaf fresh weight, root fresh weight and chlorophyll content, were recorded at the seedling stage (15 days), rapid growth stage (30 days) and maturity stage (45 days). Stem length was defined as the distance from the stem base at the soil surface to the tip of the longest fully expanded leaf. Root length was defined as the distance from the shoot–root junction to the tip of the longest primary root after careful washing of adhering soil. Leaf and root fresh weights were measured immediately after harvest using an analytical balance [21]. Chlorophyll content was expressed as SPAD values and measured using a portable chlorophyll metre (SPAD 502, Minolta, Osaka, Japan) as described by Tang et al. (2023) [21].

For antioxidant assays, fresh lettuce leaves were collected at maturity (45 days). Approximately 0.5 g of fresh tissue was ground to a fine powder in liquid nitrogen and homogenized with the extraction buffer supplied with the commercial assay kits at a ratio of 1:10 (*w/v*) under ice bath conditions. The homogenate was centrifuged at 12,000 rpm for 10 min at 4 °C, and the clear supernatant was collected for subsequent biochemical analyses [22]. Superoxide dismutase (SOD) activity was determined using a WST-8-based commercial assay kit (ADS-F-KY011-96, ADSBIO, Yancheng, China) according to the manufacturer’s instructions. The assay was conducted spectrophotometrically at 450 nm. Malondialdehyde (MDA) content was measured using a commercial kit (A003-4-1, Jiancheng, Nanjing, China). Nutritional quality was assessed by determining vitamin C (A009-1-1, Jiancheng, China), cellulose (BC4285, Solarbio, Beijing, China) and protein content (G0420F, GrisBio, Suzhou, China) following the respective manufacturer protocols.

For metabolomic analysis, frozen lettuce leaves were ground in liquid nitrogen. Approximately 0.1 g tissue was extracted with methanol, chloroform and water (2.5:1:1, *v/v/v*) containing adonitol as internal standard. After ultrasonic extraction and centrifugation at 12,000 *g* at 4 ℃, the supernatant was collected, dried under nitrogen, and derivatized with methoxyamine hydrochloride followed by N methyl N trimethylsilyl trifluoroacetamide. Samples were analyzed using GC MS (Agilent 6890A 5977A, Agilent Technologies, Santa Clara, CA, USA) equipped with an HP 5 MS column. Helium was used as carrier gas and injection was performed in splitless mode. The oven temperature was programmed from 80 to 280 degrees Celsius. Mass spectrometry was operated in electron impact mode with scan range m z 50 to 800. Metabolites were identified using the NIST 16 library with matching score above 70 percent. Quality control samples and solvent blanks were included during GC MS analysis to monitor instrument stability and potential background contamination. Multivariate analysis was conducted using SIMCA (Version 14.1) and pathway enrichment analysis was performed using MetaboAnalyst (Version 5.0) and KEGG.

### 2.5. Data Analysis and Visualization

All experiments were conducted in triplicate, and results are presented as mean values with standard deviations. One-way ANOVA was performed using IBM SPSS Statistics (Version 24.0), and statistical significance was determined at *p* < 0.05. Metabolites were retained for analysis if their matching score exceeded 70%. Pathway mapping was conducted using the KEGG database, and pathway enrichment analysis was carried out with metabolomic analysis tools.

## 3. Results and Discussion

### 3.1. Regulation of Soil Physicochemical Properties by Nanoplastics Under Arsenic Contamination

**Figure 1:**
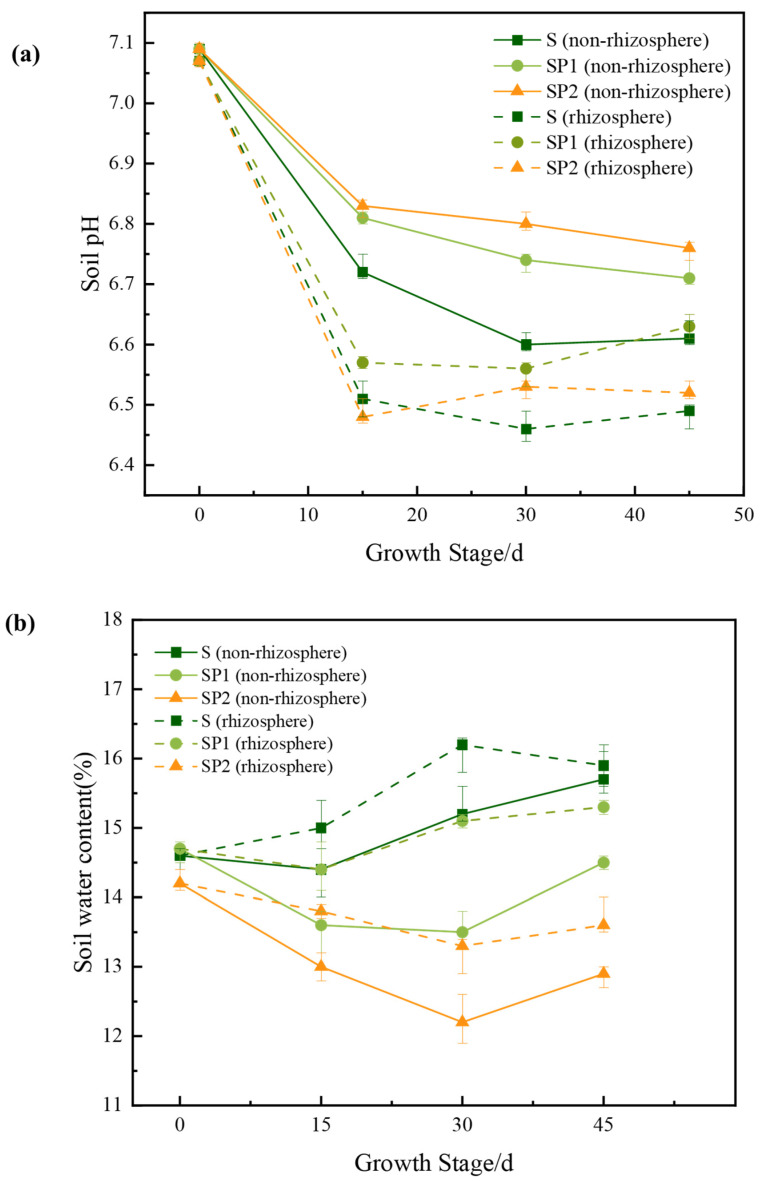
Effects of the coexistence of nanoplastics and arsenic on soil physicochemical properties in rhizosphere and non-rhizosphere compartments. (**a**) Temporal variation of soil pH under different treatments. (**b**) Temporal variation of soil water content under different treatments. Solid lines represent non-rhizosphere data, while dashed lines represent rhizosphere data. Error bars indicate standard deviations (n = 3).

Soil pH is a key factor controlling arsenic adsorption, desorption and mobility in soils. It was therefore used as the main indicator to describe how PE nanoplastics modified the soil chemical environment. Agricultural soils in Hainan and South China are generally acidic to weakly acidic according to regional surveys [23]. In this experiment, the initial soil pH was about 7.1 and declined to 6.4–6.8 during the cropping period (Figure 1a). This shift from neutral–slightly alkaline to weakly acidic conditions is consistent with soil acidification patterns reported for intensively managed croplands in southern China. In non-rhizosphere soil, pH decreased over time in all treatments, but the extent of acidification differed. The S treatment showed the largest decline, with a total drop of almost 0.5 pH units, whereas SP1 and SP2 showed smaller declines of about 0.4 and 0.3 units. This pattern indicates that PE nanoplastics mitigated non-rhizosphere soil acidification. Rhizosphere soil was more acidic than non-rhizosphere soil in all treatments, reflecting plant-driven acidification under background arsenic contamination. By Day 45, the total decline in rhizosphere pH was about 0.6 units in S, but only about 0.44 and 0.55 units in SP1 and SP2. Rhizosphere pH in SP1 was clearly higher than in S at the end of the experiment, with SP2 showing an intermediate value. These results show that PE nanoplastics not only buffered non-rhizosphere soil acidification but also partly reduced root induced acidification in the rhizosphere. The SP1 group produced the most stable buffering, helping to maintain a relatively mild yet persistent rhizosphere environment that later shaped arsenic uptake and stress responses in lettuce.

Changes in pH can influence arsenic behaviour by regulating its partitioning between soil solid phases and the soil solution through adsorption and desorption processes on mineral surfaces [24]. Microplastics and nanoplastics can influence pH by altering soil structure and the distribution of reactive surfaces. Plastic particles tend to decrease non-rhizosphere density, modify pore structure and aggregation and thereby affect the production and diffusion of carbon dioxide and organic acids [25]. In soil suspensions, PE particle surfaces can be rapidly coated by dissolved organic matter and microbial cells. These surface coatings provide additional binding sites for cations and protons and modify the competition among soil colloids, nutrient cations and H^+^ [26]. Collectively, these processes reshape proton balance and cation exchange conditions in arsenic contaminated soil. Consequently, rhizosphere acidification was weaker but remained persistent in treatments with PE nanoplastics. This pH response represents one aspect of the modified rhizosphere chemical environment under PE nanoplastics, within which arsenic uptake and stress responses in lettuce were subsequently shaped.

Soil water content influences arsenic mobility in soil and its subsequent uptake by plants, as it regulates the water availability and transport processes in the root zone. At Day 0, non-rhizosphere and rhizosphere water contents were around 14–15% in all treatments (Figure 1b), indicating similar initial moisture conditions. In non-rhizosphere soil, water content in S showed only a slight decrease by Day 15, whereas SP2 decreased by more than 1%. By Day 45, non-rhizosphere water content in S had increased from 14.6 to 15.7%, while SP2 had declined to 12.9%. This pattern shows that the high PE dose markedly enhanced non-rhizosphere water loss and restricted subsequent recovery. In the rhizosphere, overall fluctuations were smaller, but differences among treatments more clearly reflected the root-zone environment. Rhizosphere water content in S increased from 14.6 to 15.9%, whereas SP2 decreased from 14.2 to 13.6%, with SP1 consistently between these two treatments. Overall, SP2 consistently exhibited lower soil water content than S across both non-rhizosphere and rhizosphere compartments. This indicates that the high PE dose weakened soil water retention and rhizosphere buffering, keeping lettuce roots in a drier environment. These patterns in soil water content are consistent with previous studies. PE NPs have been shown to disrupt soil aggregates, modify the distribution and connectivity of soil pores, and consequently reduce soil water retention while enhancing evaporative losses [27,28]. Experimental and modelling work has shown that microplastics lower non-rhizosphere density and change pore connectivity, making water infiltration and redistribution less efficient [14,26]. In the rhizosphere, root mucilage and exudates usually help to retain water and stabilize hydraulic properties [29], which is consistent with the slightly higher rhizosphere water contents observed in S and SP1. The sustained reduction in soil water content in the SP2 treatment suggests that high PE exposure disrupts root-mediated regulation of water dynamics, resulting in decreased moisture availability in the rhizosphere. Under arsenic contaminated conditions, altered soil moisture may influence arsenic transport in the soil solution and its movement towards plant roots. However, plant water status was not directly quantified in the present study. Therefore, the mechanistic interpretation focuses primarily on arsenic transfer dynamics and stress-related physiological and metabolic responses rather than direct hydrological stress.

### 3.2. Regulation of Arsenic Migration and Accumulation by Nanoplastics in the Soil and Plant System

**Figure 2:**
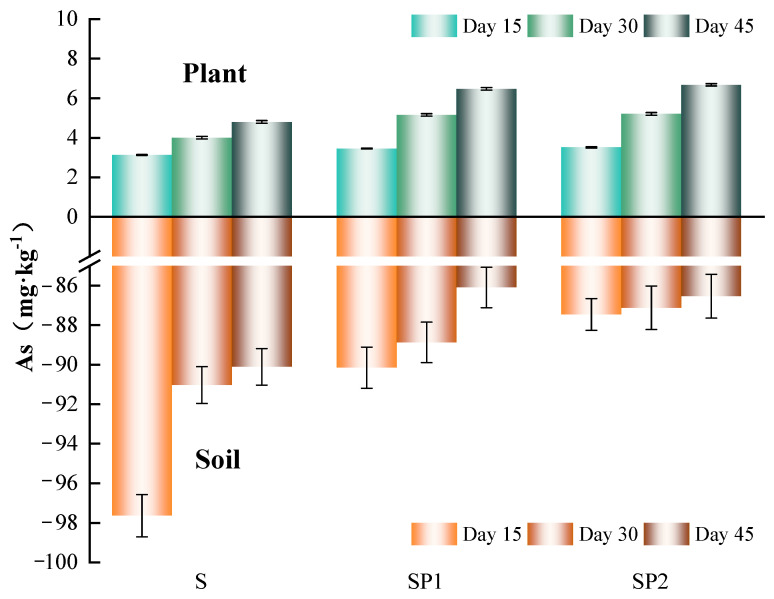
Effects of nanoplastics on arsenic concentrations in soil and plant tissues. Arsenic concentrations were measured in soil and lettuce tissues under different treatments. Soil arsenic values are plotted on the negative axis for visualization purposes to facilitate comparison with arsenic concentrations in plant tissues. Error bars represent standard deviations (n = 3).

This study used soil with a naturally high arsenic background to examine how PE nanoplastics regulate arsenic distribution and migration between soil and plants. Edible leaves were selected to characterize arsenic accumulation because leaf arsenic is directly linked to dietary risk. As shown in Figure 2, the soil arsenic concentration in the S treatment was about 98 mg·kg^−1^ at Day 15. In SP1 and SP2 it was slightly lower, with reductions of about 8–10% compared with S. This trend persisted at Days 30 and 45, and SP2 showed the largest decrease over the experimental period, being about 6.2% lower than S at the final sampling. These results indicate that PE nanoplastics mainly affected the spatial distribution of arsenic between soil solid phases and the rhizosphere microenvironment rather than causing a substantial loss of arsenic from the system. Previous studies have demonstrated that environmental exposure promotes the formation of oxygen rich functional groups on PE nanoplastics and facilitates the accumulation of dissolved organic matter. These surface features allow electrostatic interactions, hydrogen bonding and complexation with arsenic oxyanions, giving the particles an appreciable sorption capacity for arsenic [30,31].

In this study, the slightly lower soil arsenic in SP1 and SP2 than in S is consistent with such plastic, arsenic interfacial sorption and with a redistribution of arsenic among soil particles, plastic particles and rhizosphere solution. In contrast to the slight decrease in soil arsenic, PE nanoplastic treatments markedly increased arsenic accumulation in lettuce leaves. At Day 15, arsenic concentrations in SP1 and SP2 leaves were about 10–13% higher than in S. By Day 30, these increases reached about 29–30%. At the final sampling on Day 45, leaf arsenic in SP1 and SP2 was about 35% and 39% higher than in S, respectively. Over the 45-day growth period, leaf arsenic in S increased by about 53% relative to its initial value, whereas the increases in SP1 and SP2 approached 90%. Arsenic in this study originated entirely from the natural background in the field soil. However, PE nanoplastics clearly enhanced the net transfer of arsenic from soil to leaves and increased its accumulation in leaf tissue. The overall soil arsenic remained largely stable.

The different patterns observed for soil and leaf arsenic indicate that the influence of nanoplastics on arsenic toxicity is expressed mainly through changes in migration fluxes and pathways in the system. As shown above, the SP2 treatment reduced soil water content and modified pH, especially in the rhizosphere. This implies that PE nanoplastics disturbed soil aggregation, pore connectivity and water distribution, which in turn altered the partitioning conditions of arsenic between solid and solution phases [13]. In line with previous reports, PE nanoplastics can accumulate dissolved organic matter through their surface functional groups and interact with mineral colloids, thereby creating a rhizosphere microenvironment enriched in colloids and organic matter. This type of microenvironment shortens the diffusion path from soil particles to root surfaces and facilitates the local transfer of arsenic into the root zone [32].

In addition, previous studies have reported that nanoplastics can induce oxidative stress in roots, modify cell wall structure and disturb the expression of ion channels and membrane transporters, thereby enhancing root uptake of metal and metalloid elements [33]. The clear increase in leaf arsenic with increasing PE dose is consistent with these proposed mechanisms. In this system, PE nanoplastics altered the physicochemical environment of the soil and the rhizosphere. They regulated the rate at which arsenic reached root surfaces and its partitioning between solid and liquid phases, and may have also enhanced root uptake and redistribution of arsenic to leaves. Such changes in arsenic transfer should be considered when assessing environmental and dietary risks in high arsenic agricultural regions.

### 3.3. Nanoplastics Exacerbate Growth Inhibition and Morphotoxic Effects in Lettuce Grown in Arsenic Contaminated Soil

**Figure 3:**
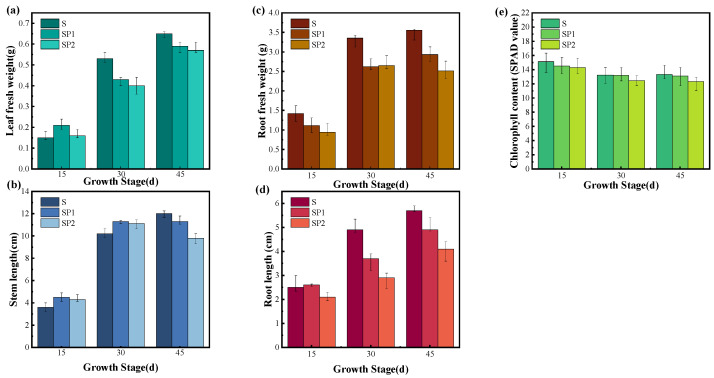
Effects of nanoplastic and arsenic co-contamination on lettuce growth performance. (**a**) Leaf fresh weight, (**b**) stem length, (**c**) root fresh weight, (**d**) root length, (**e**) chlorophyll content (SPAD value).

In naturally high arsenic soil, PE nanoplastics markedly intensified growth inhibition in lettuce. At the final harvest on Day 45, leaf fresh weight in the SP2 treatment was about 12.3% lower than in the S treatment, and stem length was about 18.3% lower (Figure 3a,b). The SPAD value in SP2 was about 7.4% lower than in S, indicating a clear reduction in leaf photosynthetic capacity (Figure 3e). These results show that, under an unchanged arsenic background, a high dose of PE nanoplastics further suppressed aboveground biomass accumulation and strengthened the toxic effect of arsenic on leaf function. Similar responses have been reported for lettuce and other crops exposed to arsenic in the presence of nanoplastics [34,35]. The root system showed an even stronger sensitivity. On Day 45, reductions in root biomass and root length in SP2 were about 29.5% and 28.1%, respectively, which were clearly larger than the reductions in leaf fresh weight and stem length (Figure 3c,d). This pattern indicates that, when PE nanoplastics were added to high arsenic soil, roots became the primary site of structural and biomass loss. Several studies on soils contaminated with metals have shown that nanoplastics often cause larger reductions in root biomass and root length than in aboveground organs. These changes are considered early indicators of declining plant vigour and limitations in water and nutrient supply [36]. The comparison between doses further indicates that the amplification of arsenic toxicity by nanoplastics depends on concentration. At the final harvest, all growth indices in the SP1 treatment fell between those observed in the S and SP2 treatments. Some indicators shifted from a slight increase at early stages to a decrease by Day 45. This behaviour suggests that a low dose of PE nanoplastics did not provide sustained mitigation of arsenic stress but gradually turned into a net inhibitory effect as exposure progressed. Similar patterns, where low doses can induce transient compensatory growth but higher doses or longer exposure lead to overall growth suppression in polluted soils, have been reported for microplastics under arsenic or metal stress [37].

When the growth phenotypes are considered together with the leaf arsenic data in Section 3.2, it becomes evident that PE nanoplastics did not attenuate arsenic toxicity in the soil with a high natural arsenic background used in this study. While the soil arsenic remained largely stable, PE nanoplastics increased the net transfer and accumulation of arsenic from soil to leaves and, at the same time, reduced root biomass and impaired leaf photosynthetic function. The combined growth and functional responses show that nanoplastics strengthened the expression of arsenic toxicity in lettuce under this background contamination. Recent work on nanoplastics in agricultural soils has similarly reported that these particles can alter pollutant partitioning between soil and plants and modulate plant stress responses. Such changes can produce more pronounced toxicity phenotypes and higher yield risks under existing contamination [38,39]. These findings indicate that, for high arsenic farmland, nanoplastics should be treated as a key contextual factor that shapes arsenic behaviour and plant toxicity in environmental and food safety assessments.

### 3.4. Oxidative Stress and Antioxidant Defence Imbalance Enhanced by Nanoplastics Under a High Arsenic Background

**Figure 4:**
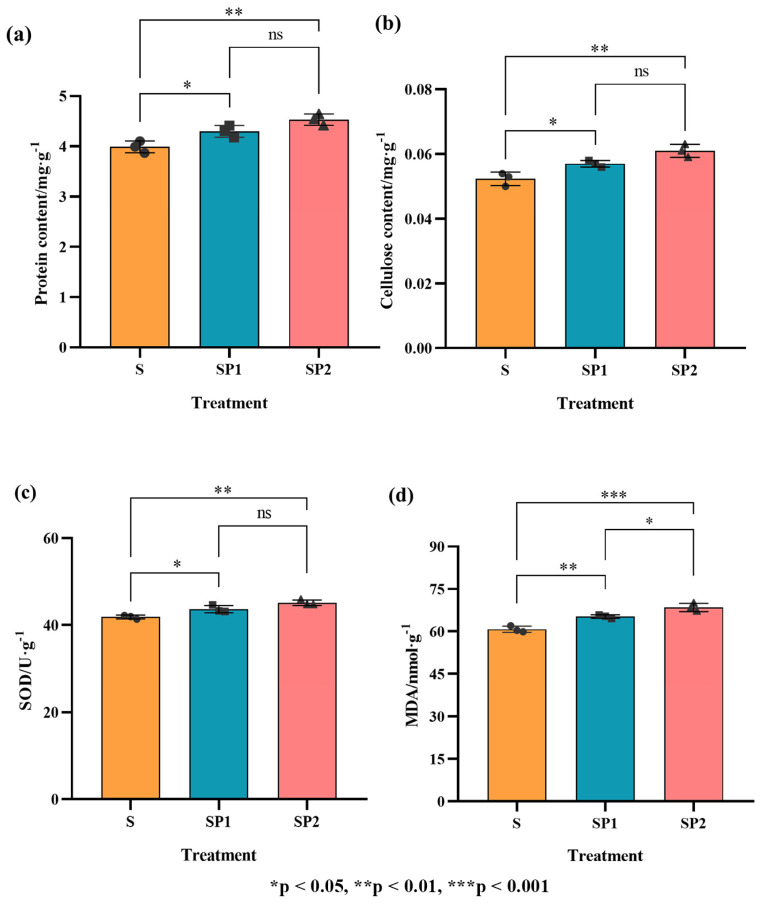
Effects of nanoplastic and arsenic co-contamination on lettuce growth indicators and oxidative stress parameters (Day 45). (**a**) Protein content in lettuce leaves. (**b**) Cellulose content in lettuce leaves. (**c**) Superoxide dismutase (SOD) activity in lettuce leaves. (**d**) Malondialdehyde (MDA) content in lettuce leaves. Error bars indicate standard deviations (n = 3). * *p* < 0.05, ** *p* < 0.01, *** *p* < 0.001; ns, not significant.

This study used lettuce leaves harvested at Day 45, when plants had reached maturity and showed high metabolic activity, to assess the effects of nanoplastics under a high arsenic background on nutritional and antioxidant indicators. As shown in Figure 4a,b, both PE nanoplastic treatments increased leaf protein and cellulose contents compared with the S treatment. Relative to S, protein content in SP1 and SP2 increased by about 8% and 14%, respectively (*p* < 0.05), and cellulose content increased by about 8% and 15% (*p* < 0.05). The presence of nanoplastics caused lettuce to allocate more resources to structural components and metabolic processes under high arsenic conditions. This response reflects reinforcement of protein pools and cell wall constituents, which helps sustain basic growth and tissue stability during prolonged stress [40,41].

To further characterize oxidative stress associated with the enhancement of arsenic toxicity by nanoplastics, superoxide dismutase (SOD) activity and malondialdehyde (MDA) content were measured in lettuce leaves (Figure 4c,d). Compared with S, SOD activity in SP1 and SP2 increased by about 7% and 13%, respectively (*p* < 0.05), indicating that in the presence of nanoplastics plants mounted a stronger antioxidant enzyme response under the same high arsenic background [42]. In parallel, MDA content also increased significantly with increasing nanoplastic dose. Relative to S, MDA in SP1 and SP2 increased by about 7% and 13% (*p* < 0.01), and SP2 was significantly higher than SP1 [22]. The sustained accumulation of MDA indicates more severe lipid peroxidation. Even though SOD activity was elevated, overall oxidative damage intensified and the antioxidant defence capacity was exceeded.

This response pattern, in which the antioxidant system is upregulated but fails to fully counter oxidative damage, is consistent with previous reports describing oxidative stress induced by nanoplastics in plants. Song et al. [43] reported that microplastic exposure markedly increases the production of reactive oxygen species in plant tissues, leading to membrane disruption and metabolic imbalance. In the present study, the addition of PE nanoplastics under the same arsenic background further enhanced superoxide dismutase related defence responses, while malondialdehyde levels also increased. This combination indicates a shift from a partially compensated oxidative condition to an imbalanced state in which antioxidant defence is insufficient. When considered together with the elevated leaf arsenic burden and the inhibition of root growth and whole plant biomass described in Section 3.2 and Section 3.3, these results indicate that nanoplastics push oxidative and antioxidant processes into a costly and inefficient operating state. This oxidative imbalance represents an important contributing mechanism that acts alongside enhanced arsenic transfer to intensify arsenic toxicity in lettuce.

### 3.5. Metabolomic Insights into Nanoplastic-Enhanced Arsenic Toxicity in Lettuce

To clarify how PE nanoplastics regulate lettuce metabolism in high arsenic soil, OPLS-DA models were used to compare the metabolic profiles of the S, SP1 and SP2 groups. The score plots showed clear cluster separation among treatments (Figure S1), and R^2^Y values of all three models were higher than 0.5, indicating good model quality for interpreting metabolic shifts [44]. In the comparison between S and SP1, SP1 samples deviated clearly from the S cluster. This pattern indicates that exposure to a low PE dose altered not only a limited number of individual metabolites but shifted the overall metabolic profile away from the arsenic only baseline [7]. In the comparison between S and SP2, the separation between groups increased further, reflecting a broader metabolic reprogramming under the higher PE dose and larger deviations from the S reference. In addition, SP1 and SP2 remained clearly separated from each other, indicating a stable metabolic response that varied systematically with PE concentration [45,46]. In this study, the OPLS DA results show that increasing PE doses progressively shift lettuce metabolism away from the arsenic background baseline and towards a stress-oriented pattern. This metabolic shift is characterized by coordinated changes in energy supply, nitrogen metabolism and antioxidant related pathways. Similar reorganization of carbon and nitrogen metabolism under combined nanoplastic and metal stress has been reported by Q. Wang et al. [47], which supports the interpretation that nanoplastics can redirect plant metabolism from a baseline condition to a stress-responsive state in contaminated soils.

**Figure 5:**
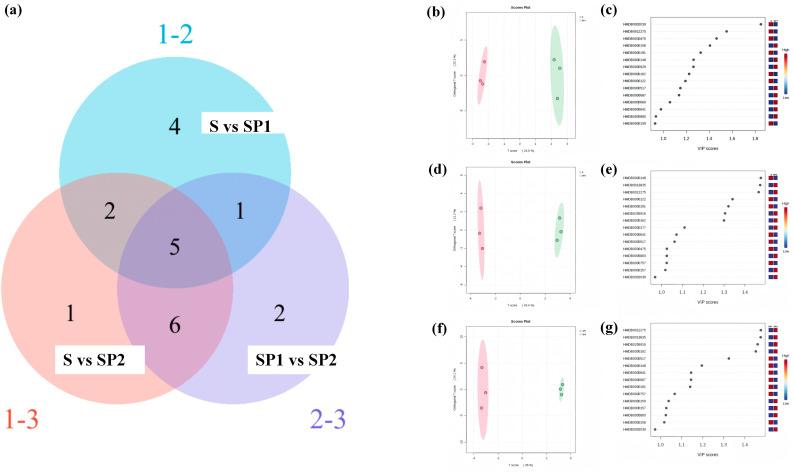

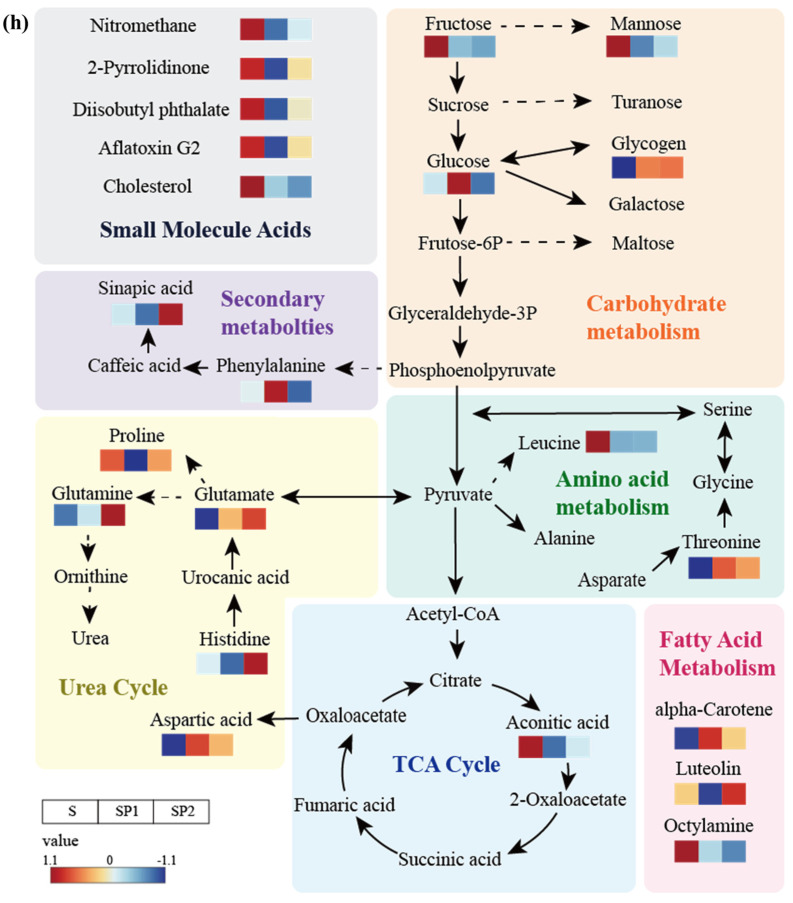
Metabolomic responses of lettuce to co-exposure of arsenate and PE nanoplastics. (**a**) Venn diagram of differential metabolites among S, SP1, and SP2 groups. (**b**,**d**,**f**) OPLS-DA score plots showing separation of metabolic profiles between treatment groups. (**c**,**e**,**g**) VIP scores of metabolites contributing to group separation in corresponding comparisons. (**h**) TCA cycle and associated metabolic pathway changes in lettuce. The relative content of metabolites is colour-coded from blue (minimum) to red (maximum) based on normalized values.

Based on these multivariate patterns, differential metabolites were screened using VIP values and their overlap among comparisons was visualized with a Venn diagram (Figure 5a; metabolite names listed in Table S1). In S vs. SP1, 2-pyrrolidinone, L-tryptophan, leucine and D-fructose were specific to this comparison. These metabolites are mainly involved in amino acid and carbohydrate metabolism, suggesting that low-dose PE already changes how primary metabolism is allocated. Tryptophan is a precursor of auxin and other signalling molecules, so its change indicates an adjustment in the supply of growth and defence signal precursors. Leucine, a branched chain amino acid, is closely linked to protein synthesis and stress responses [48]. Changes in D-fructose point to disturbed glycolysis and carbon flow, with implications for basic energy supply [49]. In S vs. SP2, L-histidine was the only specific metabolite. Histidine participates in metal chelation, antioxidant defence and pathways related to glutathione, and plays an important role in moderating toxicity under metal and oxidative stress [9]. Its enrichment at the higher PE dose indicates that lettuce reinforces metabolism related to histidine to support metal buffering and control oxidative damage as stress intensity increases [7]. In SP1 vs. SP2, cholesterol and phenylalanine were specific. Cholesterol is a precursor of plant sterols and is associated with membrane stability and signalling; shifts in its level reflect effects of PE concentration on membrane status and growth-regulation pathways [7]. Phenylalanine is a key precursor of flavonoids and lignin, so its change directly affects cell wall properties and antioxidant compound synthesis [17]. Disturbance of pathways related to phenylalanine at high PE levels indicates that nanoplastics further interfere with structural integrity and defensive secondary metabolism in lettuce exposed to arsenic stress [9].

Five metabolites were shared by all three comparisons (S vs. SP1, S vs. SP2 and SP1 vs. SP2): phenylethylamine, L-aspartic acid, glutamic acid, proline and L-arginine. These common metabolites represent core markers of metabolic reconfiguration when PE nanoplastics are added to high arsenic soil [48]. Aspartate and glutamate are key intermediates in the tricarboxylic acid cycle and nitrogen metabolism, linking carbon and nitrogen fluxes with cellular energy supply. Arginine is a central node in NO signalling, nitrogen metabolism and antioxidant regulation. Proline is a typical stress-responsive metabolite, and its level is widely used as an indicator of changes in antioxidant capacity and osmotic adjustment [17]. Phenylethylamine is related to aromatic amino acid metabolism and the formation of signalling molecules. The repeated occurrence of these metabolites shows that adding PE nanoplastics to high arsenic soil does not only affect a single pathway, but simultaneously engages carbon metabolism, nitrogen metabolism, signalling and osmotic regulation at several key junctions.

Pathway analysis further revealed a clear effect of nanoplastics that varied with dose under arsenic background conditions (Figure S2 and Figure 5h). In the comparison between SP1 and S, glycolysis and carbon metabolism pathways were perturbed, reflected by reduced D-fructose levels and a potential limitation in basic energy supply [49]. At the same time, amino acids such as tryptophan and leucine were altered, indicating that lettuce under relatively mild stress adjusts primary metabolism and the synthesis of signalling precursors. Carbon flow and protein metabolism are partially redistributed to keep a balance between growth and defence [48]. This pattern is consistent with the moderate growth inhibition observed in SP1 and indicates that PE nanoplastics at low exposure levels mainly trigger compensatory metabolic adjustments rather than an immediate breakdown of metabolic function.

In the comparison between SP2 and S, metabolic disturbance became more pronounced and reflected a broader systemic imbalance. Aconitic acid, a key TCA intermediate, decreased, pointing to reduced TCA cycle efficiency and greater pressure on cellular energy supply [9]. In contrast, glutamic acid, aspartic acid and arginine increased, indicating that pathways linked to nitrogen metabolism, NO signalling and antioxidant regulation were upregulated to cope with stronger stress [1]. Threonine also increased significantly at the high PE dose, which supports an active role of amino acid metabolism in osmotic adjustment and stress buffering. Proline was markedly higher in SP2 than in SP1, showing that lettuce relies more strongly on proline and related metabolites for osmotic protection and ROS mitigation under more severe stress [46]. Opposite to this enhancement in amino acid metabolism, phenylalanine and its derivatives, such as ferulic acid and luteolin, decreased. This indicates suppression of secondary metabolic pathways involved in ROS scavenging [7,17]. Under high PE exposure, plants increase amino-acid-based stress responses while simultaneously experiencing a limited supply of aromatic amino acids and their antioxidant derivatives. This imbalance creates a risk of insufficient antioxidant resources. The metabolomic data further indicate that, in soil with a high natural arsenic background, low PE exposure mainly results in mild metabolic perturbations characterized by compensatory reallocation of metabolic fluxes. In contrast, high PE exposure is associated with impaired tricarboxylic acid cycle function, pronounced enhancement of amino acid metabolism and suppression of secondary metabolic pathways related to antioxidant defence. When combined with the increased leaf arsenic concentrations, reduced biomass and imbalanced antioxidant status described in previous sections, these results indicate that PE nanoplastics shift the lettuce metabolic network away from an adaptive state. Under PE exposure, energy supply becomes restricted and antioxidant precursor availability declines. This metabolic transition helps explain the intensified arsenic toxicity observed in lettuce grown in high arsenic soils.

## 4. Conclusions

This study investigated how PE NPs influence arsenic behaviour and phytotoxicity in lettuce grown in agricultural soil with a naturally high arsenic background. The results show that PE NPs modified rhizosphere conditions by partially buffering soil acidification during cultivation while reducing soil water content, particularly in the rhizosphere. Although total soil arsenic concentrations changed only slightly, PE NPs altered arsenic partitioning between soil and plants and significantly increased arsenic accumulation in lettuce leaves. At the plant level, PE exposure intensified growth inhibition, reduced root biomass, root length, shoot growth and chlorophyll status, and increased oxidative stress indicators. Antioxidant responses showed elevated superoxide dismutase activity accompanied by increased malondialdehyde levels, indicating persistent oxidative damage under PE exposure. Metabolomic analysis further revealed that PE NPs reshaped metabolic regulation under arsenic stress. Low PE exposure induced moderate metabolic adjustments, whereas high PE exposure caused stronger metabolic reconfiguration associated with energy limitation and reduced antioxidant precursor supply. Overall, integrated evidence from soil properties, arsenic transfer, plant physiology and metabolomic responses demonstrates that PE nanoplastics enhance arsenic toxicity in lettuce by modifying rhizosphere conditions and promoting arsenic accumulation in edible tissues, highlighting nanoplastics as an important factor in assessing environmental and dietary risks in arsenic-affected agricultural soils.

## Data Availability

The raw data supporting the conclusions of this article will be made available by the authors on request.

## Supplementary Materials

The following supporting information can be downloaded at: https://www.mdpi.com/article/10.3390/toxics14030266/s1, Figure S1: Statistical validation parameters (R2X, R2Y, and Q2) of the OPLS-DA model used to evaluate metabolic differences among treatments; Figure S2: KEGG pathway enrichment analysis of differential metabolites under different treatments. (a) Downregulated pathways in SP1 vs S, (b) Downregulated pathways in SP2 vs S, (c) Downregulated pathways in SP2 vs SP1, (d) Upregulated pathways in SP1 vs S, (e) Upregulated pathways in SP2 vs S, (f) Upregulated pathways in SP2 vs SP1; Table S1: Classification of differential metabolites detected in lettuce leaves under PE NPs and arsenic co-exposure.
