# Supplementary material for: Polyethylene Nanoplastics Intensify Arsenic Toxicity in Lettuce by Altering Arsenic Accumulation and Stress Pathways
Source: Toxics. 2026 Mar 18;14(3):266. doi: 10.3390/toxics14030266 (PMC13029983; doi:10.3390/toxics14030266)
Supplement: Supplementary file 1 [file toxics-14-00266-s001.zip › toxics-4188776-supplementary.pdf]

Supporting Information

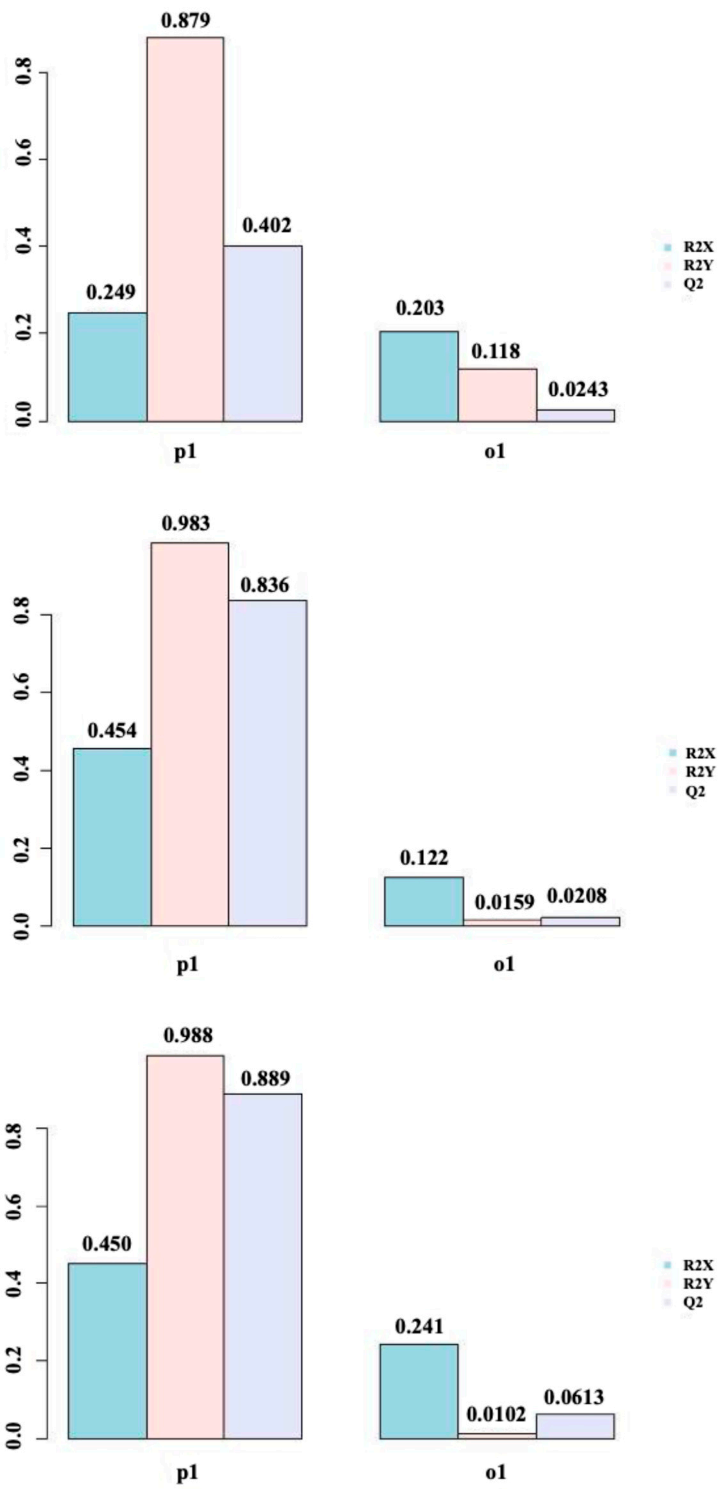

Figure S1. Statistical validation parameters (R2X, R2Y, and Q2) of the OPLS-DA model used to evaluate metabolic differences among treatments

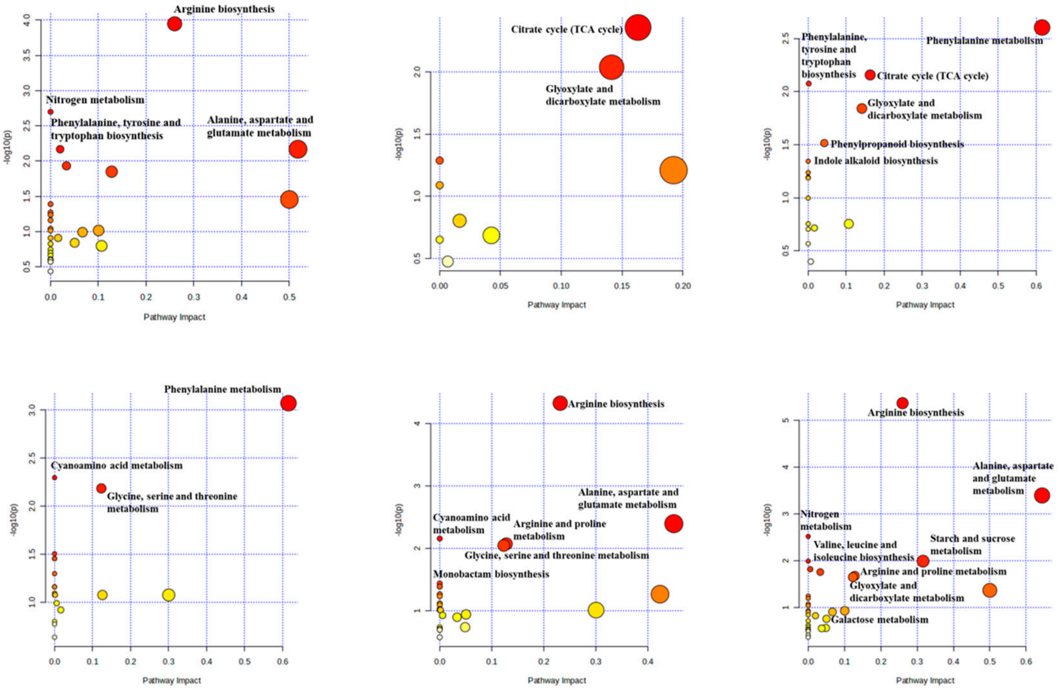

Figure S2. KEGG pathway enrichment analysis of differential metabolites under different treatments. (a) Downregulated pathways in SP1 vs S, (b) Downregulated pathways in SP2 vs S, (c) Downregulated pathways in SP2 vs SP1, (d) Upregulated pathways in SP1 vs S, (e) Upregulated pathways in SP2 vs S, (f) Upregulated pathways in SP2 vs SP1.

Table S1. Classification of differential metabolites detected in lettuce leaves under PE NPs and arsenic co-exposure

**Metabolite Name**	**HMDB ID**	**Superclass**	**Subclass**
D-Glucose	HMDB0000122	Organooxygen compounds	Carbohydrates and carbohydrate conjugates
D-Fructose	HMDB0000660	Organooxygen compounds	Carbohydrates and carbohydrate conjugates
D-Mannose	HMDB0000169	Organooxygen compounds	Carbohydrates and carbohydrate conjugates
Citric Acid	HMDB0000094	Carboxylic acids and derivatives	Tricarboxylic acids and derivatives
Cis-aconitic acid	HMDB0000072	Carboxylic acids and derivatives	Tricarboxylic acids and derivatives
Glutamic acid	HMDB0000148	Carboxylic acids and derivatives	Amino acids, peptides, and analogues
L-Arginine	HMDB0000517	Carboxylic acids and derivatives	Amino acids, peptides, and analogues
Cholesterol	HMDB0000067	Steroids and steroid derivatives	Cholestane steroids
Octylamine	HMDB0255916	Organonitrogen compounds	Amines
Diisobutyl phthalate	HMDB0013835	Benzene and substituted derivatives	Benzoic acids and derivatives
Leucine	HMDB0000687	Carboxylic acids and derivatives	Amino acids, peptides, and analogues
L-Threonine	HMDB0000167	Carboxylic acids and derivatives	Amino acids, peptides, and analogues
L-Aspartic acid	HMDB0000191	Carboxylic acids and derivatives	Amino acids, peptides, and analogues
Proline	HMDB0000162	Carboxylic acids and derivatives	Amino acids, peptides, and analogues
L-Tyrosine	HMDB0000158	Carboxylic acids and derivatives	Amino acids, peptides, and analogues
L-Tryptophan	HMDB0000929	Indoles and derivatives	Indolyl carboxylic acids and derivatives
alpha-Carotene	HMDB0003993	Prenol lipids	Tetraterpenoids
Phenylalanine	HMDB0000159	Carboxylic acids and derivatives	Amino acids, peptides, and analogues
Sinapic acid	HMDB0032616	Cinnamic acids and derivatives	Hydroxycinnamic acids and derivatives
Luteolin	HMDB0005800	Flavonoids	Flavones
Hypoxanthine	HMDB0000157	Imidazopyrimidines	Purines and purine derivatives
Glutamine	HMDB0000641	Carboxylic acids and derivatives	Amino acids, peptides, and analogues
Glycogen	HMDB0000757	Organooxygen compounds	Carbohydrates and carbohydrate conjugates
Nitromethane	HMDB0255654	Allyl-type 1,3-dipolar organic compounds	Organic nitro compounds

2-Pyrrolidinone	HMDB0002039	Pyrrolidines	Pyrrolidones
Histidine	HMDB0000177	Carboxylic acids and derivatives	Amino acids, peptides, and analogues
Phenylethylamine	HMDB0012275	Benzene and substituted derivatives	Phenethylamines
Aflatoxin G2	HMDB0030475	Coumarins and derivatives	Furanocoumarins
